# Gastric outlet obstruction due to adenocarcinoma in a patient with Ataxia-Telangiectasia syndrome: a case report and review of the literature

**DOI:** 10.1186/1477-7819-7-29

**Published:** 2009-03-12

**Authors:** Iyore A Otabor, Shahab F Abdessalam, Steven H Erdman, Sue Hammond, Gail E Besner

**Affiliations:** 1 Department of Pediatric Surgery, Nationwide Children's Hospital and The Ohio State University College of Medicine, Columbus, OH 43205, USA; 2 Department of Pediatric Surgery, Children's Hospital of Omaha, Omaha, NE 68114, USA; 3 Division of Gastroenterology, Hepatology and Nutrition, Nationwide Children's Hospital and The Ohio State University College of Medicine, Columbus, OH 43205, USA; 4 Department of Pathology and Laboratory Medicine, Nationwide Children's Hospital and The Ohio State University College of Medicine, Columbus, OH 43205, USA

## Abstract

**Background:**

Ataxia-Telangiectasia syndrome is characterized by progressive cerebellar dysfunction, conjuctival and cutaneous telangiectasias, severe immune deficiencies, premature aging and predisposition to cancer. Clinical and radiographic evaluation for malignancy in ataxia-telangiectasia patients is usually atypical, leading to delays in diagnosis.

**Case presentation:**

We report the case of a 20 year old ataxia-telangiectasia patient with gastric adenocarcinoma that presented as complete gastric outlet obstruction.

**Conclusion:**

A literature search of adenocarcinoma associated with ataxia-telangiectasia revealed 6 cases. All patients presented with non-specific gastrointestinal complaints suggestive of ulcer disease. Although there was no correlation between immunoglobulin levels and development of gastric adenocarcinoma, the presence of chronic gastritis and intestinal metaplasia seem to lead to the development of gastric adenocarcinoma. One should consider adenocarcinoma in any patient with ataxia-telangiectasia who presents with non-specific gastrointestinal complaints, since this can lead to earlier diagnosis.

## Background

Ataxia-Telangiectasia (A-T) was first reported in 1926 in three patients that presented with progressive choreoathetosis and ocular telangiectasias [1]. By 1958, it was recognized as a distinct disease process and named ataxia-telangiectasia [2]. The most common initial abnormalities noted are balance and gait problems [3]. Unsteadiness and truncal ataxia typically appear before 3 years of age, with slurred speech by 5 years of age. Patients are usually wheel-chair bound by 10 years of age due to excessive falling coupled with slow reflexes that cause serious bodily injuries. About a third of the patients present with severe immunodeficiencies accompanied by severe sino-pulmonary infections with non-opportunistic organisms, a third have moderate immunodeficiencies, and a third have no signs of immunodeficiency [4]. Serum levels of alpha fetoprotein (AFP) are usually increased in A-T patients [4-6]. There appears to be a direct correlation between serum AFP levels and age [7].

While there have been significant improvements in diagnostic modalities for A-T, there is still no cure and treatment is mainly symptomatic. Patients with A-T have a 70–250 fold increased risk for developing a lymphoreticular malignancy [8,9]. However, clinical and radiographic diagnosis of malignancy in these patients can be difficult because the presentation is usually atypical and may be confused with the infectious processes commonly associated with immunodeficiency syndromes. Six cases of gastric cancer associated with A-T have been reported in the literature in patients ages 14–26 years; of these, four cases were reported in the English literature with the last case reported in 1979 [10]. We present the case of a 20 year old female with ataxia-telangiectasia who was transferred to our institution with a diagnosis of pancreatic abnormality. Further diagnostic evaluation revealed gastric adenocarcinoma that was resected.

## Case presentation

**Figure 1:**
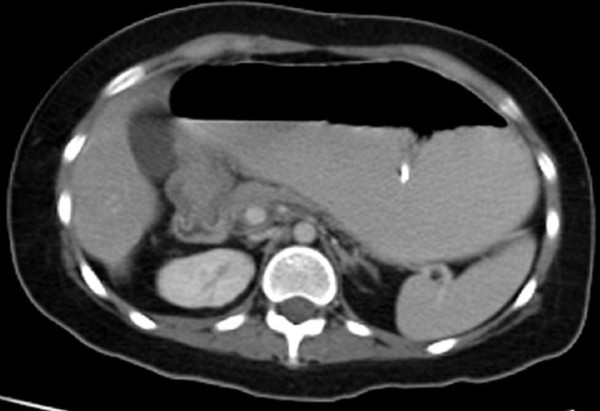
**CT scan of the abdomen with oral contrast demonstrating markedly dilated stomach, abnormal thickening in the region of the antrum and pylorus of the stomach, and adjacent pancreas thinned due to compression from dilated stomach**. There was no intra-abdominal adenopathy.

**Figure 2:**
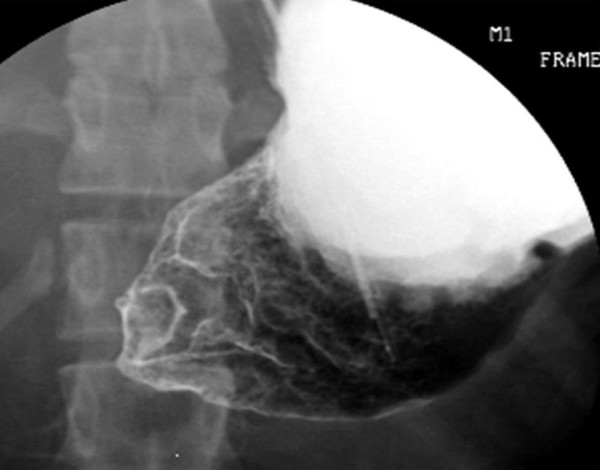
**UGI demonstrating complete gastric outlet obstruction**.

The patient is a 20 year old female that was diagnosed with ataxia-telangiectasia syndrome at 3 years of age. She had severe ataxia and was wheelchair bound by 8 years of age. She presented with a 4 week history of non-bilious emesis, early satiety, decreased appetite and a 35 lb weight loss over a one year period. The patient had undergone evaluation for these symptoms at her local hospital. A CT scan of the abdomen suggested a pancreatic mass prompting transfer to our institution. On examination, she had notable speech and cognitive delays with scleral telangiectasias, muscle wasting and other features of malnutrition. Her abdominal examination revealed a soft, non-distended abdomen with no palpable masses. Her laboratory studies were remarkable for hypokalemic, hypochloremic metabolic alkalosis, a pre-albumin level of 15 mg/dL (normal: 23–48 mg/dL), an IgG level of 461 mg/dL (normal: 546–1842 mg/dL) and an absolute lymphocyte count of 187/cu mm (normal: 1000–4800/cu mm). Other immunoglobulin levels [IgA (186 mg/dL), IgM (70 mg/dL) and IgE (<1 U/mL] were within normal limits. She was started on total parenteral nutrition (TPN) immediately upon admission. A repeat CT scan of the abdomen revealed a dilated, hypertrophied stomach consistent with chronic gastric outlet obstruction, abnormal thickening of the antrum and pylorus, and a normal appearing pancreas (Figure 1). Esophagogastroduodenoscopy (EGD) demonstrated diffuse gastritis and esophagitis with a normal appearing duodenum. She was subsequently placed on Pantoprazole^®^, Azithromycin^® ^and Metronidazole^® ^for presumed *Helicobacter pylori *infection, and kept on continuous nasogastric decompression for persistent emesis. Gastric biopsies identified non-candida fungi on the gastric epithelium and rare lymphoid aggregates in the lamina propria. A Diff-Quik stain for *Helicobacter pylori *on the biopsied specimen was negative prompting discontinuation of the triple antibody therapy. Several attempts to remove the nasogastric tube were unsuccessful, and so an upper GI series was obtained. This revealed a complete gastric obstruction (Figure 2). To address a possible submucosal infiltrative process such as lymphoma, a repeat EGD was performed to obtain deeper biopsy specimens. At this time, the scope could not be advanced into the duodenum. Deep antral biopsies identified gastric adenocarcinoma. A metastatic workup including chest CT scan and bone scan revealed no evidence of disease. After having received almost 2 weeks of TPN, she was taken to the operating room for exploratory laparotomy. Intra-operatively, she was found to have a large gastric mass involving the distal stomach which was determined to be resectable. During the dissection, there were dense adhesions between the posterior wall of the duodenum and the pancreas that were able to be divided. The tumor was resected by removing 2/3 of the distal stomach, with a minimum of 4.5 cm margins proximally and 2 cm margins distally. These margins were free of tumor on frozen and final pathologic exam. The pancreatic surface in the area of resection was biopsied in the operating room due to the finding of adhesions between the stomach and pancreas. The frozen sections showed no evidence of malignancy however, the permanent sections later revealed the presence of a focus of tumor. The patient was reconstructed with a Billroth II gastrojejunostomy after oversewing of the duodenal stump, and a feeding jejunostomy tube and Blake drain were placed. Final histologic evaluation revealed a well to moderately differentiated invasive intestinal-type adenocarcinoma of the stomach with invasion through the muscularis to the serosa, and multifocal vascular and lymphatic invasion.

Postoperatively, the patient initially did well, but then developed increased output from her abdominal drain containing high amylase and lipase levels. An UGI series revealed no evidence of anastamotic leak. On post operative day 7 she developed bloody emesis which resolved spontaneously. However, she developed increasing abdominal pain and a CT scan of the abdomen showed ascites with small bowel obstruction. She was therefore returned to the operating room for exploratory laparotomy which revealed diffuse ascites, an intact gastrojejunostomy, and an obstruction in the afferent limb of the gastrojejunostomy due to large blood clots. The clots were removed via an enterotomy in the afferent limb, and the pancreatic ascites was widely drained. She had a prolonged hospital course but recovered from surgery and was discharged to home on hospital day 48. Two months after surgery she was on continuous jejunostomy tube feeds and eating small amounts by mouth. However, three months after surgery she was refusing to eat, appeared uncomfortable although without complaints, and subsequently declined clinically thereafter. She expired 100 days from initial diagnosis while under hospice care, presumably due to wasting secondary to metastatic adenocarcinoma.

## Discussion

Our patient was the last of three children born at term to a healthy 23 year old mother. Her birth weight was 2.45 kg; there is no family history of congenital immunodeficiency disorder; her two older siblings are healthy and living. The patient was initially seen at age 18 months due to loss of previous ability to walk, poor head control and tendency to walk and fall to the right. By age 3 years, she had progressively worsening ataxia, choreoathetosis, prominent ocular telangiectasias and an elevated alpha fetoprotein of 205.4 ng/mL (normal 0–6 ng/mL) consistent with ataxia-telangiectasia. All immunoglobulin levels were within normal limits except IgM and IgE, which were slightly depressed. The patient did not receive routine immunoglobulin administration since her disorder was mostly neurologic.

Ataxia-Telangiectasia, an autosomal recessive disorder, is characterized by early onset progressive cerebellar ataxia, oculocutaneous telangiectasia, immune deficiency, and cancer. The causative gene, A-T mutated (ATM) on chromosome 11q22-23, codes for a 350 kDa Ser/Thr protein kinase that belongs to the phosphoinositide 3-kinase (PI3K)-related protein kinase (PIKK) family. ATM with associated proteins function primarily as phosphorylating agents in controlling genomic stability and regulating lymphocyte maturation. Although rare, A-T is the most common primary immunodeficiency syndrome listed in the Immunodeficiency Cancer Registry (ICR), and approximately one-third of A-T patients develop a malignancy during their lifetime. These patients most commonly develop lymphoreticular neoplasms. ATM has also been implicated in breast, lung, head and neck carcinoma, and is associated with a poor prognosis in adult patients with advanced gastric cancer [11].

**Figure 3:**
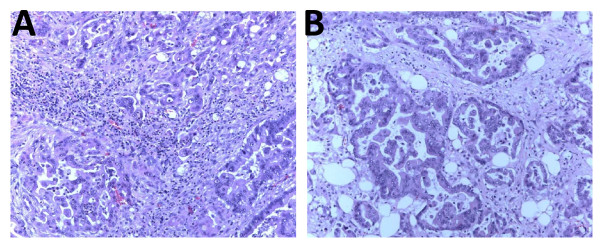
**Hematoxylin and eosin stained sections of the gastric adenocarcinoma resected from our patient**. [A] Cytological features of malignant glands; the cells are irregularly shaped with high nucleus to cytoplasm ratio and loss of nuclear polarity. The small dark cells are inflammatory cells (100× enlargement) [B] This area of tumor is in the serosa; there is redemonstration of irregular glands formed by tumor cells of varying sizes and orientation, with prominent nucleoli. The large clear spaces are fat cells (200× enlargement).

Gastric adenocarcinoma accounts for the majority of malignant gastric cancer. It arises from the glandular epithelium of the gastric mucosa. The most widely used Lauren histologic classification system divides gastric adenocarcinoma into two types – intestinal and diffuse [12]. The intestinal type, which is usually well-differentiated, originates from recognizable precancerous conditions such as gastric atrophy or intestinal metaplasia. It has a tendency to form glandular structures and spreads to distant organs hematogenously. The diffuse type is typically poorly differentiated, lacks gland formation and is composed of signet ring cells. Early metastases via lymphatic invasion commonly occur. Our patient had moderately differentiated intestinal type adenocarcinoma (Figure 3).

Six cases of gastric cancer associated with A-T have been reported in the literature in patients ages 14–26 years; of these, four cases were reported in the English literature with the last case reported in 1979 (Table 1) [13-15]. A single case series from four different hospitals revealed twelve patients with A-T and associated malignancy, with one patient that probably had gastric mucinous adenocarcinoma [16]. In the normal population, the median age for diagnosis of gastric adenocarcinoma is 70 years. Known risk factors for gastric adenocarcinoma in the general population include *Helicobacter pylori *infection, atrophic gastritis, a diet high in nitrates and salt, fried or fatty foods, low fruits and vegetables intake, smoking, male gender, and positive family history. As opposed to gastric carcinoma in the general population, gastric carcinoma in A-T patients occurs in the first or second decade of life and has an extremely poor prognosis, with the longest reported survival time of 5 months. It is important to recognize that the risk of gastric carcinoma is also elevated in patients with other primary immune deficiencies such as X-linked agammaglobulinemia, common variable immune deficiency, and X-HIGM syndromes. In patients with X-linked agammaglobulinemia, chronic atrophic gastritis, intestinal metaplasia and pernicious anemia appear to play an important role in the pathogenesis of gastric adenocarcinoma [17,18]. Thus, it is advisable to extend increased awareness of the possibility of gastric carcinoma to the entire population of primary immunodeficient patients.

**Table 1:** Previous cases of gastric adenocarcinoma associated with Ataxia-Telangiectasia Syndrome.

Authors	Symptoms on presentation	Age at diagnosis (years)/Sex	Time to death (days)
Haerer A *et al*. (1969) [13]	Nausea, intractable vomiting and weight loss	21/F	?
	
	Abdominal pain, nausea, vomiting and weight loss	19/F	90

Kondo K and Horikawa Y (1975) [14]	Severe mental retardation, gait and speech disturbances, nystagmus	21/F	?
		
		22/F	Gastric cancer confirmed at autopsy

Watanabe A *et al*. (1977) [15]	Abdominal pain, vomiting, postive fecal occult blood test	14/M	150

Frais MA (1979) [10]	Weight loss, anorexia and dyspepsia	26/M	5

Year of publication in parentheses

The A-T patients with gastric carcinoma reported to date presented with non-specific signs of abdominal pain, nausea, vomiting and weight loss. Three patients including ours had initial clinical findings suggestive of ulcer disease. On endoscopy, our patient was noted to have diffuse gastritis, which may have contributed to the development of her adenocarcinoma. All cases reported in the literature had metastatic disease at the time of exploratory laparotomy. Although depressed immunoglobulin levels are associated with increased risk of malignancy, there was no significant correlation between abnormal immunoglobulin levels and the development of gastric cancer in those patients in whom levels were checked, suggesting that injury to the gastrointestinal mucosa in the form of chronic gastritis may predispose these patients to the formation of gastric adenocarcinoma in light of their poor immunologic defense systems. Our patient further demonstrates the challenges associated with diagnosing and caring for these complex patients.

## Conclusion

Although rare, one should consider adenocarcinoma in any patient with ataxia-telangiectasia greater than 10 years of age who presents with non-specific gastrointestinal complaints, since this can lead to earlier diagnosis. Given the poor survival outcome, palliative rather than curative measures may be necessary for unresectable disease. The role of chemo-radiation therapy in A-T patients is limited as it may further predispose the patient to the risk of developing new malignancies [19]. Continuous research efforts will increase our understanding of this disease process and the role of the ATM gene in carcinogenesis.

## Consent

Written informed consent was obtained from the legal guardian for publication of this case report and accompanying images. A copy of the written consent is available for review by the Editor-in-Chief of this journal.

## Competing interests

The authors declare that they have no competing interests.

## Authors' contributions

IAO wrote the manuscript and performed the literature and case review; SFA was involved in the initial operation, performed a detailed literature review, and critically edited the manuscript; SHE performed the upper endoscopies, literature review, and critical review of the manuscript; SH was responsible for all aspects of the manuscript related to the histologic studies; GEB was involved in the patient's initial operation, and assisted in the writing and critical review of the manuscript. All authors read and approved the final manuscript.
